# Patterns of care at the end of life: a retrospective study of Italian patients with advanced breast cancer

**DOI:** 10.1186/s12904-024-01460-0

**Published:** 2024-05-22

**Authors:** Irene Giannubilo, Linda Battistuzzi, Eva Blondeaux, Tommaso Ruelle, Francesca Benedetta Poggio, Giulia Buzzatti, Alessia D’Alonzo, Federica Della Rovere, Maria Maddalena Latocca, Chiara Molinelli, Maria Grazia Razeti, Simone Nardin, Luca Arecco, Marta Perachino, Diletta Favero, Roberto Borea, Paolo Pronzato, Lucia Del Mastro, Claudia Bighin

**Affiliations:** 1 U.O. Oncologia Medica 2, IRCCS Ospedale Policlinico San Martino, Genova, Italy; 2 Department of Internal Medicine and Medical Sciences (DiMI), School of Medicine, University of Genova, Genova, Italy; 3 U.O. Epidemiologia Clinica, IRCCS Ospedale Policlinico San Martino, Genova, IT Italy; 4 U.O.C. Clinica Di Oncologia Medica, IRCCS Ospedale Policlinico San Martino, Genova, Italy; 5 SSD Cure Palliative Ed Hospice, IRCCS Ospedale Policlinico San Martino, Genova, Italy

**Keywords:** End of life, Palliative care, Breast cancer

## Abstract

**Objectives:**

To better understand the type of care offered to Italian patients with advanced breast cancer at the End-of-Life (EoL), we conducted a retrospective observational study. EoL was defined as the period of six months before death.

**Methods:**

One hundred and twenty-one patients with advanced breast cancer (ABC) treated at IRCCS San Martino Policlinic Hospital who died between 2017 and 2021 were included. Data about patient, disease, and treatment characteristics from breast cancer diagnosis to death, along with information about comorbidities, medications, imaging, specialist evaluations, hospitalization, palliative care and home care, hospice admissions, and site of death were collected.

**Results:**

98.3% of the patients received at least one line of active treatment at EoL; 52.8% were hospitalized during the selected period. Palliative (13.9%), psychological (7.4%), and nutritional evaluations (8.2%) were underutilized. Palliative home care was provided to 52% of the patients. Most of the patients died at home (66.1%) and fewer than one out of five (18.2%) died at the hospital. Among the patients who died at home, 27.3% had no palliative support.

**Conclusions:**

Our findings indicate that palliative care in EoL breast cancer patients is still inadequate. Only a minority of patients had psychological and nutritional support While low nutritional support may be explained by the fact that typical symptoms of ABC do not involve the gastrointestinal tract, the lack of psychological support suggests that significant barriers still exist. Data on the site of death are encouraging, indicating that EoL management is increasingly home centered in Italy.

## Introduction

In recent years, the prognosis of advanced cancer care has been dramatically changed, especially thanks to the new available treatments that induce durable responses and improve overall survival [1, 2]. Most advanced solid tumors, however, are still incurable, and palliative care remains a mainstay of cancer patient care [2, 3], with multiple randomized trials demonstrating that, when integrated into cancer care, it improves patients’ quality of life (QoL) and symptom control [3, 4], reduces aggressive end-of-life (EOL) care [5–8] and diminishes psychological distress both for patients and informal caregivers. Finally, it decreases health care costs [9]. 

Although these benefits are especially significant for patients nearing the end of their life, palliative and end-of-life (EoL) care are still insufficiently applied, as demonstrated by inappropriate hospitalizations and insufficient referral to hospice [10, 11]. This may be due to various factors, including a lack of palliative care specialists and services, patients’ and families’ expectations, and healthcare professionals’ inadequate communication skills [12, 13]. Novel therapeutic options may be part of the problem, as they have generated a whole new set of treatment options in advanced stages, as well as improvements in terms of QoL [14], also causing an increase in patient and family expectations.

Adequate use of palliative care services depends also on the ability of healthcare professionals to estimate and communicate patients’ prognoses. Indeed, accurate prognostic estimates allow clinicians to refer patients for palliative and EoL care earlier within the disease trajectory [15]. Studies have also shown that when patients at the end of life have an accurate perception of their prognosis, they are less likely to seek active treatment [16, 17]. These realities hold true for patients with advanced breast cancer, although prognostic estimations are difficult in this population due to heterogeneity in disease subtypes [9, 18].

A better understanding of utilization patterns of anticancer drugs and other healthcare resources at the end of patients’lives may facilitate shared decision-making on starting or discontinuing active treatment [19]. Thus, it may lead to improved EoL care and QoL in patients with advanced breast cancer, as well as enable health systems to plan and allocate resources properly. Nevertheless, there is a paucity of information in literature about the use of healthcare resources for patients with specific cancer types, including breast cancer, during the EoL [19]. Therefore, the aim of this retrospective observational study was to evaluate the use of healthcare resources in the last six months of life of advanced breast cancer patients.

## Materials and methods

### Patients

We selected patients with advanced breast cancer who were treated at IRCCS Policlinico San Martino Hospital and died of this disease between 2017 and 2021.

Eligible patients were identified within the Gruppo Italiano Mammella (GIM) 14/BIOMETA study (ClinicalTrials.gov Identifier: NCT02284581), which has been collecting data about all the advanced breast cancer patients that started treatment at our hospital since 2015.

Inclusion criteria were: i) advanced breast cancer diagnosis, ii) date of death between 2017 and 2021, iii) availability of information about the patient management during the last six months of life.

**Fig. 1:**
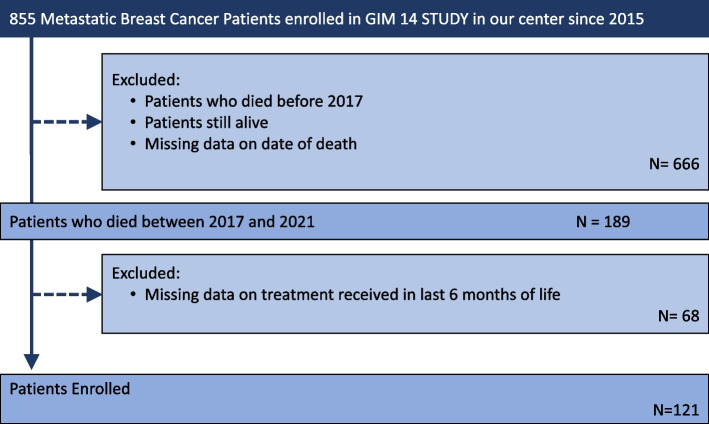
Enrolment algorithm. How we selected patients with metastatic breast cancer enrolled in GIM 14 study since 2015

Overall, the database included 855 patients; of these, 666 did not qualify for the study as they had died before 2017 or were still alive at the time of selection. Among the remaining 189 patients, 68 lacked information on the treatment received during the last six months of life (mostly due to difficulties in retrieving data from paper records used before 2019), resulting in 121 patients eligible for the present study (Fig. 1).

### Data collection and definitions

Data about patients, disease, and treatment characteristics from breast cancer diagnosis to death, were collected from hospital records, along with information about comorbidities, medications used, imaging, specialist evaluations, hospitalization, emergency department admission, palliative care team intervention, palliative home care, admission to hospice and site of death. All breast cancer subtypes were included.

We defined palliative care interventions as interventions in the hospital environment performed by our palliative care team, composed of palliative medicine physicians and nurses, both for hospitalized patients and for outpatients. For palliative home care, we define a team, also composed of palliative medicine physicians and nurses, that performs palliative interventions at the patient’s home.

EoL was defined as the period of six months before death. This interval was chosen according to the longer expected overall survival of advanced breast cancer if compared to other tumor types.

Luminal A disease was defined as Estrogen and Progesterone receptor positivity (positive nuclear staining of ≥ 10%), HER2 negativity, and protein Ki67 < 20%. Luminal B disease was defined as Estrogen and/or Progesterone receptor positivity (positive nuclear staining of ≥ 10%), HER2 negativity, and protein Ki67 ≥ 20%. HER2 positivity was defined as an immunohistochemistry score of 3 + , or 2 + with a positive fluorescence in situ hybridization (FISH) result. Triple-negative breast cancer (TNBC) was defined as a tumor with Estrogen and Progesterone negativity (positive nuclear staining of < 10%) and HER2 negativity.

### Outcomes

Outcomes of interest were the line of therapy ongoing at the time of death, emergency department admissions, hospital units where patients were admitted when hospitalized, specialist evaluations, radiotherapy, and site of death. Furthermore, possible associations between healthcare resources and individual characteristics of patients (i.e., concomitant medications, age at death, type of breast cancer, and sites of metastases), were investigated.

### Statistics

Descriptive statistics were used to describe the frequency of healthcare use. Logistic regression was used to identify predictors of healthcare use in the last six months of life. A *p*-value of ≤ 0.05 was considered statistically significant. An Odds Ratio (OR) ≥ 1 indicates a predictor of increased healthcare use. A Confidence Interval over 95% was considered statistically significant.

## Results

### Study population

Patient characteristics are summarized in Table 1. Treatment and evaluations received in the EoL period are summarized in Table 2.

**Table 1:** Characteristics of the enrolled patients

Characteristics	*N* = 121 (%)
Median age at early breast cancer diagnosis (years, range)	57 (31–81)
Median age at metastatic cancer diagnosis (years, range)	64 (31–88)
Median age at death (years, range)	68 (32 – 88)
Comorbidities	
No	27 (22.3)
Yes	94 (77.7)
Cardiovascular comorbidities	70 (57.8)
Endocrinologic comorbidities	28 (23.1)
Psychiatric comorbidities	28 (23.1)
Concomitant medications	
No	5 (4.1)
Yes	116 (95.9)
Cardiovascular therapy	72 (59.5)
Steroid therapy	75 (62.0)
Antalgic therapy	106 (87.6)
Breast cancer subtype	
Luminal A	34 (28.1)
Luminal B	53 (43.8)
Her2 +	17 (14.0)
TNBC	17 (14.0)
Visceral involvement at diagnosis	25 (20.7)
Encephalic involvement at diagnosis	3 (2.5)
Visceral involvement at death	103 (85.1)
Encephalic involvement at death	31 (25.6)

**Table 2:** Antineoplastic treatment received in the last six months of life

Treatment received in the last 6 months of life	*N* = 121 (%)
Chemotherapy need	
No	30 (24.8%)
Yes	91 (75.2%)
Chemotherapy line number	
1	12 (13.2%)
2	16 (17.6%)
3	21 (23.1%)
4	17 (18.7%)
5	5 (5.5%)
6	11 (12.1%)
7	4 (4.4%)
8	2 (2.2%)
9	2 (2.2%)
10	1 (1.1%)
Hormonal therapy need	
No	66 (54.5%)
Yes	55 (45.5%)
Hormonal therapy line number	
1	16 (29.1%)
2	22 (40%)
3	7 (12.7%)
4	4 (7.3%)
5	1 (1.8%)
6	1 (1.8%)
7	4 (7.3%)
Anti Her2 therapy need	
No	108 (88%)
Yes	13 (12%)
Anti Her2 therapy line number	
1	2 (15.4%)
2	0 (0%)
3	11 (84.6%)
Radiotherapy need	
Yes	32 (73.5%)
No	89 (26.5) %)

#### Diagnosis of breast cancer and metastasis

Among the 121 patients with advanced breast cancer included, all were female and the median age at primary disease diagnosis was 57 years (range 31–81); 28.1% of the patients were diagnosed with luminal A breast cancer (*n* = 34), 43.8% with luminal B (*n* = 53), 14.1% with HER2 + (*n* = 17) and 14% with TNBC (*n* = 17). Median age at initial diagnosis of metastasis was 64 years (range 31 – 88), and median age at death was 68 years (range 32—88). Patients younger than 60 years old at death were 37 (30.6%). The year of initial diagnosis of metastasis was between 2002 and 2021; 20.7% had visceral involvement at the initial diagnosis of metastasis (*n* = 25) and 2.5% had brain metastasis (*n* = 3).

#### Active cancer treatments

In terms of active treatments, we considered the last line of therapy received in the last six months of life. Fifty-four patients (44.6%) received combination treatment, more specifically: chemotherapy associated with hormonal therapy for 30 luminal patients (24.8%); chemotherapy associated with anti-HER2 agents for 13 HER2-positive patients (10.7%); hormonal therapy and biological therapy for 11 patients (9.1%). In detail, 75.2% of the patients (*n* = 91) received chemotherapy during the last six months of their life, 45.4% hormonal therapy (*n* = 55), and 10.7% anti-HER2 therapy (*n* = 13). Among the patients who received chemotherapy, for 13.2% (*n* = 12) it was the 1st line of treatment, for 59.4% (*n* = 54) it was the 2nd to 4th (for 17.6% the 2nd, for 23.1% the 3rd, for 18.7% the 4th), and for 27.4% (*n* = 25) it was the 5th to the 10th.

Among the receptor-positive patients, 55 received hormonal therapy during the last six months of life; for 29.1% (*n* = 16) it was the 1st line of treatment, for 60% (*n* = 33) the 2nd to 4th, and for 10.9% (*n* = 6) it was the 5th to the 7th.

Among the 17 HER2-positive patients, 13 received targeted HER2 treatment in the last six months of life; for eight of these 13 patients (61.5%) it was the 1st line of treatment, for five (38.5%) it was the 2nd or later line.

Overall, 119 out of 121 patients (98.3%) received at least one line of active treatment in the last six months of life. Among the patients who did not receive any active treatment in the last six months of life, one patient refused treatment due to personal reasons while the family refused treatment for the other patient, who was frail and no longer competent owing to cognitive deterioration.

#### Radiotherapy

Radiotherapy was administered to 32 patients (26.4%); bone and brain were the most common sites of irradiation (50%, *n* = 16, and 40.6% *n* = 13, respectively).

#### Comorbidities

We defined comorbidities as any distinct additional medical condition that had existed or occurred during the clinical course of our patients [20]. Overall, cardiovascular comorbidities were the most common (57.8%, *n* = 70), followed by endocrine and psychiatric comorbidities (both 23.1%, *n* = 28). In total, 94 patients had at least one comorbidity; 21/94 of these patients (22.3%) were younger than 60 years old.

#### Concomitant medications

We defined concomitant medications as any other prescription medications or drugs that the study participant took in the evaluated period of time, in addition to the anti-cancer therapy. Almost all of the patients (95.9%, *n* = 116) used non-cancer medication. This included steroids (62%, *n* = 75), cardiovascular drugs (59.5%, *n* = 72), psychiatric drugs (32.2%, *n* = 39), endocrine drugs (22.3%, *n* = 27), and neurological drugs (16.5%, *n* = 20). These patient groups were heterogeneous in terms of both age and breast cancer subtype.

#### Imaging

Most of the patients (81.8%, *n* = 99) had at least one computed tomography scan (CT) during the last six months of their life, with a median interval from the last CT scan to death of 46 days (range 1–182 days); among these, 26 patients had a CT scan in the last month, while eight patients had at least one CT scan in the last week. Twenty-two patients had no CT scans at EoL. However, eight of these had a different type of imaging test (four had at least one Positron Emission Tomography (PET) scan and one ultrasound, one had one PET scan, and three had at least one ultrasound). The 14 patients who had no imaging were heterogeneous in terms of both age and breast cancer subtype. Details about imaging are reported in Table 3.

**Table 3:** Radiological evaluations received in the last 6 months of life

Radiological Evaluations received in the last 6 months of life	*N* = 121 (%)
CT scan	
0	22 (18.2)
1	26 (21.5%)
2	42 (34.7%)
3	21 (17.4%)
4	4 (3.3%)
5	5 (4.1%)
6	1 (0.83%)
PET	
0	107 (88.4%)
1	10 (8.26%)
2	2 (1.65%)
3	2 (1.65%)
Bone Scan	
0	117 (96.7%)
1	4 (3.3%)
UltrasoundE	
0	76 (62.8%)
1	30 (24.8%)
2	10 (8.3%))
3	1 (0.8%)
4	2 (1.6%)
5	2 (1.6%)

#### Specialist evaluations (other than palliative)

Most of the patients (83.4%, *n* = 101) had at least one oncological evaluation during the observed period, with a median interval from the last oncological evaluation to death of about two months (59 days). Among other specialized evaluations, the most common were those with a physical medicine specialist (17.3%, *n* = 21), a cardiologist (16.5%, *n* = 20), or a neurologist (15.7%, *n* = 19). Nutritional and psychological evaluations were performed in 7.4% and 8.2% of patients, respectively (*n* = 9 and *n* = 10). Four of the nine nutritional evaluations were performed in young patients (age < 60 yrs.), as were six of the ten psychological evaluations. Details about evaluations are shown in Tables 4 and 5.

**Table 4:** Specialist evaluations received in the last 6 months of life

Specialist evaluations in the last 6 months of life	*N* = 121 (%)
Specialist evaluations received	
Yes	109 (90.1%)
No	12 (9.9%)
Oncological evaluations	
0	20 (16.5%)
1	28 (23.1%)
2	37 (30.6%)
3	26 (21.5%)
4	8 (6–6%)
5	1 (0.8%))
6	1 (0.8%)
Cardiological evaluations	
0	101 (83.5%)
1	16 (13.2%)
2	2 (1.6%)
3	1 (0.8%)
4	1 (0.8%)
Neurological evaluations	
0	102 (84.3%)
1	13 (10.7%)
2	5 (4.1%)
13	1 (0.8%)
Infectious disease evaluations	(0.8%)
0	109 (90%)
1	5 (4.13%)
2	5 (4.13%)
3	1 (0.83%)
6	1 (0.83%)

**Table 5:** Supportive care evaluations received in the last six months of life

Specialist evaluations in the last 6 months of life	*N* = 121 (%)
Palliative evaluations	
0	104 (85.9%))
1	11 (9.1%)
2	2 (1.6%)
3	2 (1.6%)
4	2 (1.6%)
Nutritional evaluations	
0	112 (92.6%)
1	7 (5.8%)
2	2 (1.6%)
Psychological evaluations	
0	111 (91.7%)
1	9 (7.4%)
3	1
Physical medicine evaluations	
0	100 (82.6%)
1	18 (14.8%)
2	2 (1.65%)
3	1 (0.83%)

#### Hospitalizations

Roughly half of the patients (52.8%, *n* = 64) were hospitalized during the last six months of life, mostly at the Medical Oncology Unit (40.4%, *n* = 49) and approximately a month before death (median 32.7 days before death, range 1–157 days).

#### Emergency department admissions

Fifty-two patients (43%) were admitted to the emergency department during the last six months of life. The median interval between the last admission and death was 35.5 days (range 0–181 days), without significant differences by age or breast cancer subtype (Table 6).

**Table 6:** Hospital and emergency department admissions in the last six months of life

Number of Hospital and Emergency department admissions	*N* = 121 (%)
Ordinary hospital department admission	
Yes	64 (52.9%)
No	57 (47.1%)
Oncology Unit admission	
0	72 (59.5%)
1	41 (33.9%)
2	5 (4.1%)
3	2 (1.6%)
4	1 (0.8%)
Cardiology Unit admission	
0	120 (99.2%)
1	1 (0.8%)
Neurology Unit admission	
0	118 (97.5%)
1	3 (2.5%)
Emergency Department admission	
0	69 (57%)
1	37 (30.6%)
2	13 (10.7%)
3	1 (0.8%)
4	1 (0.8%)

#### Palliative care interventions and palliative home care

Palliative care interventions were performed in 13.9% of the patients (*n* = 17). Among these, 11 (64.7%) were younger than 60 years old. Palliative home care was provided to 63/121 patients (52%), without significant differences by age or breast cancer subtype.

#### Site of death

Most of the patients died at home (66.1%, *n* = 80); among these patients, a substantial proportion (27.3%, *n* = 33) had no palliative support. Thirteen patients (10.7%) died in hospice, twenty-two died at the hospital (18.2%): 14 (11.6%) at the Medical Oncology Unit, 6 (5%) at the emergency department, and 2 (1.6%) at the Internal Medicine Unit. Six patients died elsewhere (e.g., at a nursing home).

The group of patients who died at the hospital showed no significant difference from patients who died elsewhere in terms of age or breast cancer subtype.

**Fig. 2:**
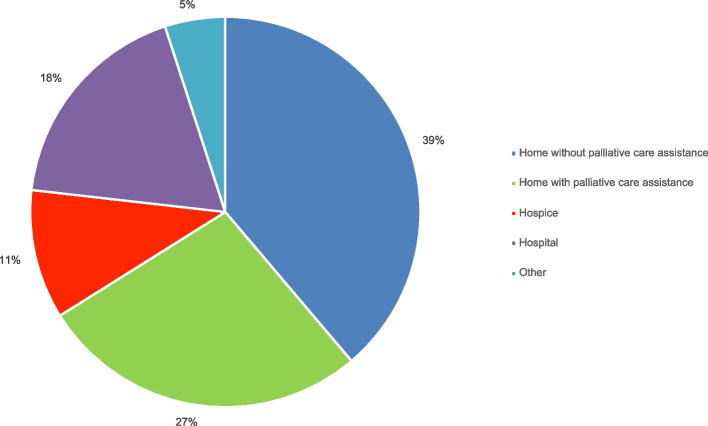
Site of death

Most of the patients who received palliative home care died at home (47 out of 63, 74.6%), five died in Hospice (7.9%), seven at the Hospital (11.1%), and four at various other locations (6.3%). Sites of death are shown in Fig. 2.

#### Predictors of healthcare use

Comorbidities and concomitant medications were the factors most closely associated with the number of CT scans received (OR 4.02, 95% CI 1.5–10.8; *p* = 0.006; OR 7.66 1.2–49 *p* = 0.03, respectively).

Cardiovascular comorbidities (OR 3.00, 95% CI 1.32–6.5; *p* = 0.005), endocrine comorbidities (OR 4.14, 1.33–12.91; *p* = 0.006), concomitant steroids (OR 2.52, 95% CI 1.16–5.45; *p* = 0.018) and visceral involvement at metastatic diagnosis (OR 0.26, 95% CI 0.10–0.65; *p* = 0.035) were associated with a higher number of specialist evaluations.

Hospitalization was most frequently associated with comorbidities (OR 2.29, 95% CI 0.95–5.54; *p* = 0.06), specifically cardiovascular (OR 1.98, 95% CI 0.95–4.11; *p* = 0.06) and endocrine comorbidities (OR 2.78, 95% CI 1.11–6.95; *p* = 0.023). Association with concomitant steroids was also observed (OR 3.33, 95% CI 1.55–7.19; *p* = 0.002).

Emergency department admission was most frequently associated with comorbidities, particularly cardiovascular (OR 2.32, 95% CI 1.09–4.93; *p* = 0.027) and endocrine (OR 3.12, 95% CI 1.29–7.54; *p* = 0.009).

Hospitalization, emergency admissions, and specialist evaluations were not associated with the age of the patient nor with a specific breast cancer subtype. The same was true for CT scans. Palliative care interventions and nutritional and psychological evaluations were more common among younger patients but not associated with any specific breast cancer subtype.

Overall, comorbidities seemed to be the most relevant factor in terms of predicting healthcare use.

## Discussion

In this retrospective observational study, we attempted to gain a more in-depth understanding of the type of care offered to patients with advanced breast cancer in the EoL period.

With cancer patients being actively treated close to the end of their life, the expectation is that a subset of these patients will be hospitalized for symptom relief and due to treatment-related toxicities [21]. We observed that roughly half of the patients in our study (52.8%) were hospitalized during the last six months of life, mostly at the Medical Oncology Unit (40.4%) and about one month before they died. Although cross-study comparisons should be taken with caution, we note that this frequency is lower than reported in other studies on patients with advanced breast cancer. Schmitz et al., for instance, found a higher admission rate of 76% within 6 months of death [19], while Tanguy-Melac and colleagues found a 90% hospital admission rate in an advanced breast cancer population over 12 months [22]. While the former study reported that age < 65 years, de novo metastatic breast cancer, and a survival time < 1 year were associated with hospital admissions within six months of death, in our study, these were most frequently associated with comorbidities and concomitant medications, particularly steroids. This finding may be explained by the fact that steroid use is widespread among patients with brain metastases and/or liver failure, and/or respiratory distress. However, the comparatively lower frequency of hospitalizations seen in our population is encouraging.

Another consequence of using active treatment in the last months of life is delayed referral for palliative care in situations where early palliative care is not the standard approach, owing to misconceptions about its appropriate integration in the management of patients with advanced breast cancer [23]. Indeed, we found concerning evidence of insufficient access to palliative care, which confirms findings from previous Italian reports [5, 24]. Palliative home care was provided to just over half of the patients (52%), and palliative care evaluations were performed in 13.9% of the patients.

Several studies have shown that multiple barriers exist to palliative care referral. Hui and colleagues identified a range of barriers to delivering timely palliative care [25]. One key barrier is stigma, as many oncologists still perceive that palliative care is only appropriate for patients at the very end of their lives and that a referral is likely to reduce patients’ hope [5]. This type of perception may be especially common among oncologists involved in advanced breast cancer, given the prognostic difficulties and multiple treatment options available, as discussed earlier. Another barrier is the issue of inconsistent referral criteria. Studies have found that among patients with advanced disease, those with solid tumors and younger age had greater palliative care [5, 26]. This is confirmed by our findings, as most of the patients in our study who received a palliative care evaluation (64.7%) were younger than 60 years old. Overall, palliative care referral often takes place in a non-systematic way, with many patients who could benefit from it being referred only in the latest stages of the disease or not at all [23, 27]. This points to the importance of hospital-based referral criteria, developed jointly by oncologists and palliative care specialists in accordance with locally available resources [25].

Our results highlight the lack of psychological and nutritional support available for advanced breast cancer patients at our hospital, as only 7.4% and 8.2% of our patients had psychological or nutritional evaluation during the observed period, respectively. While low nutritional support is somewhat to be expected, given that typical symptoms of advanced breast cancer tend not to involve the gastrointestinal tract, the lack of psychological support suggests that significant barriers, such as poor screening and low understanding and acceptability of psychology and mental health services still exist, leading to scant referrals and low uptake [28].

Most of the patients in our study died at home (66.1%, *n* = 80); this finding is substantially higher than the 37% average rate reported for the general Italian population by the Italian National Institute of Statistics in its latest available report (year 2018). At the same time, the 18.2% frequency of hospital deaths we found is much lower than the rate reported for Italian cancer patients in a cross-national European end-of-life study, in which hospital death rates were lowest for Dutch patients (28%), and highest for Italian patients (39%). It is also lower than the rate reported by a recent Dutch study on healthcare use during the last six months of life in patients with advanced breast cancer (25%) [19]. In addition, a population-based study on intensity of care, expenditure, and place of death of French women with breast cancer found that almost 70% of these patients died at hospital [29]. The authors noted that few data are available in the literature on the site of death of breast cancer patients specifically. They quoted two studies conducted in the United States, one on a cohort of 123 patients [30], and the other on a cohort of 947 patients [31]. In the first study, 53% of the patients died at home, in the second, 25% died at the hospital. Comparing sites of death across studies can be challenging, as variations in observed frequencies may depend on how cases are defined and how EoL care is organized. Our findings may be explained by the fact that EoL management for patients with breast cancer is increasingly home-centered in Italy, also because these patients often eventually die of liver failure, which, unlike respiratory failure, for instance, does not prompt admission to the emergency department or hospitalization. Moreover, considering their relatively young median age, it is possible that a subset of our patients had a physically fit relative who could take care of them.

Overall, concomitant medications and especially comorbidities seemed to be the main predictors of healthcare use among patients in our study. Previous studies have shown that comorbidities increase the toxicity of specific treatments, increase hospitalizations, create difficulties with treatment, and lead to higher healthcare costs [32], but little evidence seems to be available about how these factors interact and play out in the specific context of patients with advanced breast cancer at the end of their life.

Our study has some limitations. First, it is a retrospective analysis of a relatively small number of patients from a single institution. Moreover, the patients included were treated over a rather extended period of time, in an era when approaches to the treatment of advanced breast cancer were changing and medical records went from paper to electronic. Finally, we cannot rule out that the results were biased by the fact that over 1/3 of patient records were incomplete and could not be included in the analysis.

To overcome these biases, we are now conducting a prospective study, selecting breast cancer patients who are not expected to survive beyond six months due to their tumor type, treatment outcomes, and cancer localization. Based on the results of this prospective study we plan to establish a true simultaneous care pathway with the palliative team at our hospital, increasing the involvement of psychologists and developing training efforts aimed at improving the awareness among oncologists of the need to prevent overuse of health care services and unwanted or inappropriate care near the EoL. Explorations will also be conducted to assess the satisfaction of informal caregivers (after patients have passed away) with the palliative support provided.

## Data Availability

The datasets used and analyzed during the current study are available from the corresponding author upon reasonable request.
